# Accurate prediction of protein–ligand interactions by combining physical energy functions and graph-neural networks

**DOI:** 10.1186/s13321-024-00912-2

**Published:** 2024-11-04

**Authors:** Yiyu Hong, Junsu Ha, Jaemin Sim, Chae Jo Lim, Kwang-Seok Oh, Ramakrishnan Chandrasekaran, Bomin Kim, Jieun Choi, Junsu Ko, Woong-Hee Shin, Juyong Lee

**Affiliations:** 1 Arontier Co., 241, Gangnam-daero, Seocho-gu, Seoul, 06735 Republic of Korea; 2 Department of Molecular Medicine and Biopharmaceutical Sciences, Graduate School of Convergence Science and Technology, Seoul National University, Seoul, 08826 Republic of Korea; 3 Data Convergence Drug Research Center, Korea Research Institute of Chemical Technology, Daejeon, 34114 Republic of Korea; 4 Department of Medicine, Korea University College of Medicine, Seoul, 02841 Republic of Korea; 5 Research Institute of Pharmaceutical Science, College of Pharmacy, Seoul National University, Seoul, 08826 Republic of Korea; 6 College of Pharmacy, Seoul National University, Seoul, 08826 Republic of Korea

**Keywords:** Virtual screening, Protein–ligand binding prediction, Hit discovery, Protein–ligand docking, Graph neural network, Protein–ligand binding pose prediction, Deep-learning, Physics-based scoring function

## Abstract

We introduce an advanced model for predicting protein–ligand interactions. Our approach combines the strengths of graph neural networks with physics-based scoring methods. Existing structure-based machine-learning models for protein–ligand binding prediction often fall short in practical virtual screening scenarios, hindered by the intricacies of binding poses, the chemical diversity of drug-like molecules, and the scarcity of crystallographic data for protein–ligand complexes. To overcome the limitations of existing machine learning-based prediction models, we propose a novel approach that fuses three independent neural network models. One classification model is designed to perform binary prediction of a given protein–ligand complex pose. The other two regression models are trained to predict the binding affinity and root-mean-square deviation of a ligand conformation from an input complex structure. We trained the model to account for both deviations in experimental and predicted binding affinities and pose prediction uncertainties. By effectively integrating the outputs of the triplet neural networks with a physics-based scoring function, our model showed a significantly improved performance in hit identification. The benchmark results with three independent decoy sets demonstrate that our model outperformed existing models in forward screening. Our model achieved top 1% enrichment factors of 32.7 and 23.1 with the CASF2016 and DUD-E benchmark sets, respectively. The benchmark results using the LIT-PCBA set further confirmed its higher average enrichment factors, emphasizing the model’s efficiency and generalizability. The model’s efficiency was further validated by identifying 23 active compounds from 63 candidates in experimental screening for autotaxin inhibitors, demonstrating its practical applicability in hit discovery.

**Scientific contribution**

Our work introduces a novel training strategy for a protein–ligand binding affinity prediction model by integrating the outputs of three independent sub-models and utilizing expertly crafted decoy sets. The model showcases exceptional performance across multiple benchmarks. The high enrichment factors in the LIT-PCBA benchmark demonstrate its potential to accelerate hit discovery.

**Supplementary Information:**

The online version contains supplementary material available at 10.1186/s13321-024-00912-2.

## Introduction

Predicting the binding affinities of protein–ligand complexes is crucial in computer-aided drug discovery (CADD). A drug candidate must have selectivity to a target, strong binding affinity with the target while weak affinities with other proteins. Experimental determination of binding affinity is both difficult and time-consuming, forming a bottleneck in drug discovery [1]. Predicting binding affinity primarily aids in identifying potent binders from virtual molecule libraries and accurately determining a ligand’s binding pose within a protein pocket. Correctly predicting affinity helps select promising candidates and understand ligand–protein interactions, which is crucial for designing optimized ligands.

Molecular dynamics (MD)-based methods like MM-GB/SA, MM-PB/SA, RBFE, and absolute binding free energy calculations are recognized for their accuracy in predicting protein–ligand binding affinity and have gained significant popularity [2–5]. The accuracy of these methods varies depending on their computational complexities, with RBFE achieving approximately 1 ~ 2 kcal/mol [6], while MM-PB/SA typically exhibits an error of a range of 2–3 kcal/mol or even worse [3, 7]. Despite their high predictive accuracy, MD-based methods require substantial computational resources and time for generating MD trajectories, making them unsuitable for ultra-large-scale virtual screening, particularly when dealing with billions of chemicals [8, 9].

In contrast, traditional protein–ligand docking calculations are computationally faster but generally less accurate in predicting binding poses. Scoring functions in traditional docking are generally categorized into three types. The first category, the physics-based scoring function, adopts functional forms and atomic parameters derived from force fields. For instance, the scoring function employed in AutoDock4 [10] combines terms related to van der Waals interactions, hydrogen bonding, electrostatic potential, and solvation to calculate the binding affinity. Other physics-based empirical scoring functions of DOCK [11] and AutoDock [10, 12] are also widely used. The second scoring function category in protein–ligand docking is empirical scoring functions, which approximate protein–ligand interactions using simplified formulas [13] for computational efficiency. They generally incorporate terms considering van der Waals interactions, hydrogen bonding, electrostatic interactions, and other affinity-related factors. Several well-known empirical scoring functions are X-Score [14], PLP [15], ChemPLP [16], and GlideScore [17]. The third category is the knowledge-based scoring function derived from a structural database of protein–ligand complexes [18, 19]. These scoring functions assume that atomic pairs occurring more frequently in the database contribute more to the overall binding affinity than less frequent pairs. Knowledge-based scoring functions examine the frequencies of atom pairs and their corresponding distances from a sizeable structural database, such as the Protein Data Bank (PDB). These frequencies are then transformed into pseudo potentials using the inverse-Boltzmann equation. Well-known examples of this category are DrugScore [20] and PMF [21]. Overall, the Pearson correlation coefficients of traditional docking scoring functions between predicted binding affinities and experimental values range from 0.2 to 0.5 [22].

Machine learning (ML) methods, particularly deep learning, have gained significant traction to balance between accuracy and computational efficiency in estimating binding affinity. Several programs, such as K_DEEP_ [23], OnionNet [24], and Pafnucy [25], employed convolutional neural networks (CNN). In these approaches, a protein–ligand complex structure is represented as a 3D grid, with each voxel point mapping to specific atom types within the complex. By using the 3D grid representation as input, the models were trained to predict binding affinities. Similarly, we developed AK-Score [26], a binding affinity prediction method utilizing 3D CNNs. Most models showed high correlations with experimental values with Pearson correlation coefficients of over 0.8 [27]. This high accuracy demonstrates the efficacy of using deep learning techniques for affinity prediction.

In addition to CNN, graph neural networks (GNN) have emerged as another powerful approach for estimating binding affinity. GNN represents a molecule as a connected graph. Generally, atoms are considered nodes, and atom pairs covalently bonded or spatially close are considered edges. Unlike CNN-based models, GNN-based models are translationally and rotationally invariant. Various GNN-based binding affinity prediction methods have been published [28–30], including InteractionGraphNet [31], GraphBAR [32], and SS-GNN [33]. These GNN-based methods achieved high Pearson correlation coefficients ranging from 0.78 to 0.87, demonstrating the effectiveness of GNN in capturing the complex interplay between atoms and accurately estimating binding affinities.

However, it was observed that these ML-based binding affinity prediction models were not successful in virtual screening [34]. The low screening power was attributed to the limitations of the training sets and uncertainties in docking poses. The ML-based scoring functions showed low accuracy when novel or highly dissimilar proteins to the training sets were shown. In addition, identifying near-native bound conformations is essential to resolve the pose uncertainties caused by docking. Lim and coworkers developed the PIGNet model based on a physics-based scoring function with machine-tuned parameters [35]. For PIGNet, many decoy conformations were used together with the crystal structures, and the model was trained to make the binding energies of decoys higher than the crystal structures. Shen and coworkers introduced RTMScore [36], which predicts the residue–atom distance likelihood potential using a mixture density model [37]. The benchmark results show that PIGNet and RTMScore are highly efficient in identifying hit molecules.

In this study, we propose a new protein–ligand interaction prediction model, AK-Score2, aiming for more accurate virtual screening. AK-Score2 consists of three independent sub-networks: AK-Score-NonDock, AK-Score-DockS, and AK-Score-DockC. The three sub-networks were trained differently; each model predicts the interaction probability between a protein and ligand, the binding affinity and root mean square deviation (RMSD) of a complex separately, and a penalized binding affinity by predicted RMSD. AK-Score2 considers the error of binding affinity and the RMSD of predicted poses. These factors are incorporated into the loss functions of the model by using numerous native-like and decoy conformations.

Additionally, AK-Score2 performs the final prediction by combining the outputs from the three models and a physics-based scoring function. We found that combining ML and physics-based scoring functions yields the best performance. We benchmarked AK-Score2 using three independent decoy sets: CASF-2016 [13], DUD-E [38], and LIT-PCBA [39]. The results demonstrate that our model outperforms existing methods in hit screening. The performance of AK-Score2 is benchmarked with three decoy sets for hit screening: Throughout the benchmarks, AK-Score2 shows improved performance, and higher enrichment factors, than most state-of-the-art methods for forward screening. Additionally, we performed experimental validation of the performance of AK-Score2 by screening hit candidates of autotaxin [40] (ATX). We generated 63 novel inhibitor candidates of ATX using our MolFinder approach [41]. The experimental validation, the synthesis of the selected compounds followed by kinetic assays, confirmed 23 of 63 molecules as active, representing a success rate significantly surpassing conventional hit discovery paradigms.

## Methods

### Training dataset

Protein–ligand complex structures with experimentally determined binding affinity data (K_i_/K_d_) from the general set of PDBbind v2020 [42] were used as a training set in this study. The training set included the general set, while the redundant samples in the core set of PDBbind were removed from the training set. Additionally, samples that were not docked when using AutoDock-GPU [43] were excluded (Table 1). To reduce the computational cost, we converted the proteins into binding pockets, defined as the residues within 5.0 Å around the crystallized ligands. We excluded complexes whose ligand binding pockets were not recognized by the RDKit toolkit [44].

**Table 1:** The summary of training and the benchmark data

Training data	Protein–ligand complex (crystal-native)	Conformational decoy	Cross-docked decoy	Random decoy
PDBbind general set [59] (without CASF2016 [13])	17,225	900,910	1,720,958	1,721,583

Benchmark test data	Protein	Active	Inactive
CASF2016 coreset	285		
CASF2016 docking data	285	5100	16,865
CASF2016 screening data	57	27,839	1,561,424
DUD-E [38]	102	22,886	1,144,300
LIT-PCBA [39]	15	501,500	139,750,100

The PDBbind general set, excluding CASF-2016, was used as the training dataset and three data-augmented datasets: native, conformational decoy, and cross-docked decoy sets. The CASF-2016 coreset, DUD-E, and LIT-PCBA were used as benchmark test datasets

To ensure efficient training, we created four different types of complex structure datasets: a native set $\mathcal{N}$, a conformational decoy set ${\mathcal{D}}_{\text{conf}}$, a cross-docked decoy set ${\mathcal{D}}_{\text{cross}}$, and a random decoy set ${\mathcal{D}}_{\text{random}}$. ${\mathcal{D}}_{\text{conf}}$ were generated by redocking the native ligand to the native binding pocket. We used AutoDock-GPU [43] to generate decoy structures by sampling 50 poses around the native binding pose of the ligand using a 22.5 Å × 22.5 Å × 22.5 Å size grid box, resulting in 900,910 complex poses. For some targets, not enough diverse poses were generated, or all docked poses had RMSD values less than 2 Å. For such cases, we generated additional 900 poses to ensure maximum diversity. The resulting poses were clustered by AutoDock-GPU, up to 100 for each target. The RMSD distributions of ${\mathcal{D}}_{\text{conf}}$ are displayed in Supplementary Fig. 1.

The cross-docked set, ${\mathcal{D}}_{\text{cross}}$, was generated by randomly selecting 100 ligands (excluding a sample’s native ligands) from other complexes of PDBbind and redocking them to create 1,720,958 complexes. Additionally, we created a random decoy set ${\mathcal{D}}_{\text{random}}$, to cover more expansive chemical space. We randomly sampled 100 ligands from the Enamine Discovery Diversity Set (DDS-50) [45] and docked them to the proteins in PDBbind, resulting in 1,721,583 complexes.${\mathcal{D}}_{\text{cross}}$ ${\mathcal{D}}_{\text{random}}$.

Random ligands may act as strong binders to novel proteins, potentially resulting in false-negative predictions by our model. However, the success rate of conventional high-throughput screening is notably low, typically ranging from 0.001 to 0.151% [46, 47]. For training the AK-Score model, we randomly selected 100 compounds per protein, resulting in an expected occurrence of less than one false-positive per protein. Therefore, we assumed that the impact of false-negative samples to be minimal and unlikely to significantly affect the model’s performance.

### Benchmark test dataset

The CASF-2016, DUD-E [38] and the LIT-PCBA [39] datasets were used to benchmark our models (Table 1). The CASF2016 dataset consists of 285 complexes with docking decoys, i.e., conformational decoys, to assess the accuracy of near-native conformation identification, and screening decoys, i.e., cross-docked decoys, to evaluate the accuracy of discriminating non-binders from true binders. In the CASF-2016 set, the docking decoys are generated by redocking the native ligands of the 285 complexes, and their RMSD values range from 0 to 10 Å. Conformations whose RMSD is less than 2 Å are considered native. The screening decoys were generated by docking 285 ligands on 57 proteins, each with 100 poses. Each of the 57 proteins had five of the 285 active ligands. We used 285 native conformations for scoring and ranking, 21,465 conformations for docking, and 1,589,263 conformations for the screening benchmark. We created a graph representation of a ligand binding pocket for each target. We excluded the conformations that were not recognized by the RDKit toolkit.

Additionally, we utilized the LIT-PCBA dataset, which was compiled from high-confidence PubChem bioassay data [39]. This dataset comprises 15 proteins, with 9780 active and 407,839 unique inactive ligands. We generated each ligand’s lowest energy 3D conformer using RDKit to prepare the ligands for docking. Since LIT-PCBA contained multiple receptor PDBs per target, we selected the structure with the best resolution. Each protein’s active and inactive ligands were re-docked using AutoDock-GPU, resulting in 50 poses for each target, leading to 489,000 active and 20,391,950 inactive docked conformations. For each target, the best-scored conformation was used for benchmarking. The RTMScore results were retrieved from reference [36].

The performance of screening methods was estimated with the enrichment factor (EF) [48]. The enrichment factor is defined: $EF=\frac{{N}_{\text{experimental}}^{x\%}}{{N}_{\text{expected}}^{x\%}}=\frac{{N}_{\text{experimental}}^{x\%}}{{N}_{\text{active}}\cdot x\%}$, where ${N}_{\text{experimental}}^{x\%}$ is the number of experimentally verified hits among the top *x*% scored molecules and ${N}_{\text{active}}$ is the total number of hit molecules in a dataset.

### Model overview

**Fig. 1:**
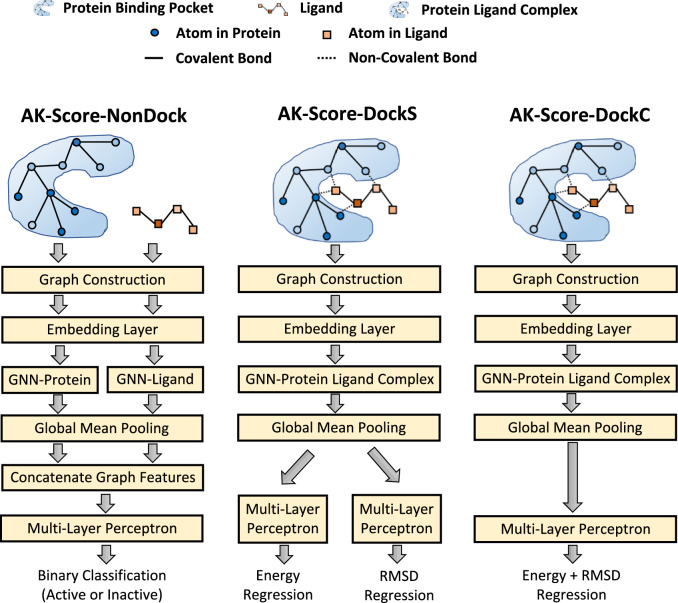
Model Architectures of three individual models consisting of AK-Score2: AK-Score-NonDock, AK-Score-DockS, and AK-Score-DockC. Three different models based on GNN were developed for predicting binding affinity. AK-Score-NonDock model inputs a pair of protein binding pockets and ligand separately, without considering their binding pose, to predict their interaction probability. AK-Score-DockS and AK-Score-DockC models input a protein–ligand complex that has a binding pose to predict energy (kcal/mol) and RMSD (Å) or their combination for further estimating binding affinity

In this work, we developed three independent networks: AK-Score-NonDock, AK-Score-DockS, and AK-Score-DockC (Fig. 1). The AK-Score-NonDock model received a protein binding pocket and a ligand conformation file as separate inputs, and outputs an interaction probability, performing a binary classification. The AK-Score-DockS model accepts a protein–ligand complex structure and, in turn, provides a predicted binding free energy and an RMSD to a crystal structure separately. The AK-Score-DockC model also received a protein–ligand complex bound structure as an input. However, its output is a single energy value, penalized by adding its predicted RMSD to a crystal structure. A more comprehensive description of our methodology is provided in the subsequent section.

### Graph construction

The models’ inputs are undirected graphs $G=(V,E)$, where $V$ denotes a group of nodes and a node represents a heavy atom in a protein or a ligand, where $E$ denotes a group of edges that links nodes with interactions. The node and edge features are described in Supplementary Table 1 and 2, respectively, and the detailed feature construction approach is described in Supplementary Method S1. We defined two edge types: a covalent bond and a non-covalent bond. Covalent bond edges were generated between all covalently bonded heavy atom pairs. Non-covalent bonds were constructed between pairs of protein and ligand atoms with a distance less than 8 Å, a threshold commonly used in molecular mechanics force fields. For example, in molecular dynamics simulations using the particle mesh Ewald method, van der Waals and electrostatic interactions within an 8–10 Å cutoff are calculated in real space, while those beyond, weaker interactions, are treated in Fourier space. Thus, we assumed that 8 Å was a reasonable threshold for capturing significant non-covalent interactions. To summarize, the dimensions of node and edge feature vectors of a graph for AK-Score-DockS and AK-Score-DockC are 73 and 24. Those of a graph for AK-Score-NonDock are 73 and 12 because they have only covalent bond edges.

### Model architecture

The initial node features pass an embedding layer, a simple linear layer projecting 73-dimensional node feature vectors to 256-dimensional node embedding vectors for all three models. The enriched node embeddings and edge features are put into GNN layers to update node information. All the models are constructed by stacking multiple GATv2 [49] layers, implemented in the PyTorch Geometric library [50] with default settings.

For AK-Score-NonDock, two separate GNNs, GNN-Protein and GNN-Ligand, accept a ligand binding pocket graph and a ligand graph, respectively (Fig. 1). AK-Score-DockS and AK-Score-DockC receive a graph representing a protein–ligand complex structure containing non-covalent bonds. The GNNs that process a protein–ligand complex and a protein structure consist of five layers of GATv2 [49]. The GNN for processing a ligand includes three layers of GATv2 as a ligand has much fewer atoms and bonds than protein structures. Each layer of GATv2 is followed by a ReLU activation layer [51] and a dropout [52] layer sequentially.

After updating node and edge feature vectors through the GNN layers, a global mean pooling layer is applied to aggregate all node and edge information. For AK-Score-NonDock, a binding pocket embedding and a ligand embedding vectors are concatenated to form a single combined graph embedding. Afterward, an embedding vector passes a multi-layer perceptron module to output a single value corresponding to the objective value of a GNN. The multi-layer perceptron module comprises three linear layers with a ReLU activation [51] and a dropout [52] layer. The detailed hyperparameters of the network structure of the models are described in Supplementary Table S3.

### Model output and loss function

#### AK-Score-NonDock model

The AK-Score-NonDock model is designed to estimate the interaction probability between a binding pocket and a ligand in a binary fashion, active or inactive. Binary cross entropy (BCE) was used as a loss function ${L}^{ND}$:

1 $${L}^{ND}= \text{BCE}({p}_{\text{pred}}, { p}_{\text{true}})$$

where ${L}^{ND}$ denotes the loss function, ${p}_{\text{pred}}$ is the predicted interaction probability, and ${p}_{\text{true}}$ represents ground truth. The ${p}_{\text{true}}$ of the native protein–ligand complexes in PDBbind is unity, and those of the complexes in ${\mathcal{D}}_{\text{cross}}$ are zero. ${\mathcal{D}}_{\text{conf}}$ was not used to train this model because the model does not consider bound poses. The model training integrated conformations from $\mathcal{N}$, ${\mathcal{D}}_{\text{cross}}$, and ${\mathcal{D}}_{\text{random}}$, incorporated in a 1:2:2 ratio per batch. For inferential purposes, a sigmoid function was applied to ensure ${p}_{\text{pred}}$ values fall within the range of 0 to 1, where a higher value denotes a greater interaction probability.

#### AK-Score-DockS model

The AK-Score-DockS model receives a protein–ligand complex structure as input and predicts the binding free energy and the RMSD from the native structure separately. AK-Score-DockS has a common GNN module followed by two separate multi-layer perceptron (MLP) modules. The common GNN module consists of an initial embedding layer, five GATv2 layers, and a global mean pooling layer. The critical aspect of the AK-Score-DockS model is that different loss functions are applied conditionally according to the nature of the conformational sets. We used three conformational sets to predict binding free energy prediction: $\mathcal{N}$, ${\mathcal{D}}_{\text{cross}}$, and ${\mathcal{D}}_{\text{random}}$.

If an input structure is from $\mathcal{N}$, the loss function, ${L}_{\text{native}}^{E}$, is defined as the mean squared error (MSE) between the experimental and predicted binding free energies, ${E}_{\text{true}}$ and ${E}_{\text{pred}}$:

2 $${L}_{\text{native}}^{E}=\text{ MSE}\left({E}_{\text{pred}}, { E}_{\text{true}}\right)$$

We assumed that randomly docked compounds to target proteins are highly likely to be inactive [46, 47]. Conventionally, compounds with IC_50_ values in the range of 1–10 μM are considered hits, while inactive molecules are typically associated with IC_50_ values exceeding 100 μM. A binding affinity of −5.0 kcal/mol corresponds to an IC_50_ value of approximately 240 μM, supporting our assumption that such compounds are likely to be inactive. Thus, we hypothesized that the pairs in ${\mathcal{D}}_{\text{cross}}$ and ${\mathcal{D}}_{\text{random}}$ have weaker binding affinities than a threshold value, −5.0 kcal/mol. Thus, inspired by PIGNet [35], the loss function for these decoys, ${L}_{\text{cross}\_\text{random}}^{E}$, is designed to predict their binding affinities higher than the threshold:

3 $${L}_{\text{cross}\_\text{random}}^{E}= \text{max}(-{E}_{\text{pred}}-5. 0)$$

In other words, if ${E}_{\text{pred}}$ is lower than the threshold, the difference is regarded as a loss. On the contrary, if ${E}_{\text{pred}}$ is higher than the threshold, the loss is zero because it is considered correct.

For RMSD prediction, we used $\mathcal{N}$ and ${\mathcal{D}}_{\text{conf}}$ datasets. If an input is included in $\mathcal{N}$, the loss function, ${L}_{\text{native}}^{\text{RMSD}}$, is defined as the deviation from zero, which is the ground truth value of the native conformations:

4 $${L}_{\text{native}}^{\text{RMSD}}= \text{MSE}\left({R}_{\text{pred}}, 0\right),$$

where ${R}_{\text{pred}}$ is predicted RMSD value. The samples from ${\mathcal{D}}_{\text{conf}}$ were generated by redocking a native ligand to a protein binding pocket. Thus, the loss function for ${\mathcal{D}}_{\text{conf}}$,${L}_{\text{conf}}^{\text{RMSD}}$, is defined as the MSE between the true and predicted RMSD values:

5 $${L}_{\text{conf}}^{\text{RMSD}}= \text{MSE}\left({R}_{\text{pred}},{ R}_{\text{true}}\right),$$

where ${R}_{\text{true}}$ and ${R}_{\text{pred}}$ are the true and predicted RMSD values from the native pose.

The final total loss of AK-Score-DockS, ${L}_{\text{total}}^{\text{S}}$, is the weighted sum of the four losses defined above:

6 $${L}_{\text{total}}^{\text{S}}= {L}_{\text{native}}^{E}+ 5{L}_{\text{cross}\_\text{random}}^{E}+{L}_{\text{native}}^{\text{RMSD}}+{L}_{\text{conf}}^{\text{RMSD}}.$$

During training, the conformations from $\mathcal{N}$, ${\mathcal{D}}_{\text{conf}}$, ${\mathcal{D}}_{\text{cross}}$, and ${\mathcal{D}}_{\text{random}}$ were fed into the model in a 1:4:2:2 ratio per batch. Note that the conformations from ${\mathcal{D}}_{\text{conf}}$ were not considered for calculating loss functions related to binding free energy because their binding free energies are unknown. Similarly, the conformations from the non-binding decoy sets, ${\mathcal{D}}_{\text{cross}}$ and ${\mathcal{D}}_{\text{random}}$, were not used to calculate RMSD losses because they have no true binding poses to calculate RMSD. The predicted RMSD value is considered a penalty during inferencing, meaning a high RMSD means a wrong binding pose. Consequently, the two separately predicted values are summed up to obtain the final energy to evaluate the model.

#### AK-Score-DockC

The AK-Score-DockC model has a similar architecture to AK-Score-DockS but produces a single combined value for binding affinity and RMSD predictions. A predicted binding free energy is penalized with a predicted RMSD. The loss functions for $\mathcal{N}$, ${\mathcal{D}}_{\text{cross}}$, and ${\mathcal{D}}_{\text{random}}$ are identical to those of AK-Score-DockS (Eqs. 7 and 8). The critical difference of AK-Score-DockC is that, for the conformational decoys, it predicts the penalized binding affinity, the sum of experimental binding affinity ${E}_{\text{true}}$ and the RMSD from the crystal structure, ${R}_{\text{true}}$ (Eq. 9). Since the conformations in ${\mathcal{D}}_{\text{conf}}$ do not have ground truth binding free energy, we estimated the value by adding one’s RMSD to the experimental binding affinity ${E}_{\text{true}}+{ R}_{\text{true}}$; we assumed that the binding free energy would deteriorate as the conformation deviates from the native state.

The final total loss of the AK-Score-DockC model, ${L}_{\text{total}}^{\text{C}}$ is the sum of the above three losses (Eqs. 7 ~ 9): ${L}_{\text{native}}^{\text{E}}$, ${L}_{\text{cross}\_\text{random}}^{\text{E}}$, ${L}_{\text{conf}}^{\text{E}}$. Note that we weighted ${L}_{\text{cross}\_\text{random}}^{\text{E}}$ by a factor of 5.

7 $${L}_{\text{native}}^{\text{E}}= \text{MSE}({E}_{\text{pred}},{E}_{\text{true}})$$

8 $${L}_{\text{conf}}^{\text{E}}= \text{MSE}({E}_{\text{pred}},{E}_{\text{true}}+{R}_{\text{true}})$$

9 $${L}_{\text{cross}\_\text{random}}^{\text{E}}= \text{max}(-{E}_{\text{pred}}-5, 0)$$

10 $${L}_{\text{total}}^{\text{C}}= {L}_{\text{native}}^{\text{E}}+ {L}_{\text{conf}}^{\text{E}}+5{L}_{\text{cross}\_\text{random}}^{\text{E}}$$

### Training detail and ensemble strategy for evaluation

Given the limited number of true binding samples, we generated a larger set of non-binding decoy samples, using a higher ratio of decoys to improve the model’s ability to identify true negatives and reduce false positives. For the AK-Score-NonDock and AK-Score-Dock models, the ratios 1:2:2 and 1:4:2:2, respectively, were tuned by testing different configurations (e.g., 1:3:3, 1:4:4, 1:2:1:1, 1:6:3:3) and monitoring validation loss and performance during the initial training epochs. In summary, the conformations from $\mathcal{N}$, ${\mathcal{D}}_{\text{conf}}$, ${\mathcal{D}}_{\text{cross}}$, and ${\mathcal{D}}_{\text{random}}$ were fed into the model in a 1:4:2:2 ratio per batch.

All models were developed with the PyTorch [53] and PyTorch Geometric library [50]. They were trained for 1000 epochs using the Adam optimizer [54] with a learning rate of 0.0001, and a dropout rate for all dropout layers was set to 0.1. The batch size was 30 for AK-Score-NonDock and 36 for AK-Score-DockS and AK-Score-DockC. Hyperparameters, including embedding length and the number of GAT layers, were optimized through tuning. Embedding lengths (128, 256, 512) and GAT layers (3, 5, 7) were evaluated, with final selections based on validation set performance. Additional tuning included learning rate, optimizer, and dropout rate. Due to resource constraints, models were not fully trained for all hyperparameter combinations; instead, early validation loss and accuracy were used to identify the most promising configurations.

The ensemble strategy was explored for better performance. The AK-Score-NonDock accepts a binding site and ligand conformations separately and outputs their interaction probability from 0 to 1. AK-Score-DockS and AK-Score-DockC, receive a protein–ligand complex structure and predict real-valued energy. The non-docking and docking-based models were ensembled by multiplying the outputs of these models. The rationale of this approach is that if either of the models mispredicts a given complex structure, the final prediction becomes close to zero, corresponding to non-binding. Namely, our model predicts binding only when the two independently trained models yield similar results.

Furthermore, inspired by the Onion-SFCT model [55], we combined the physics-based AutoDock-GPU scoring function ${E}_{\text{ATD}}$, with our deep-learning-based predictions. We call the final two models AK-Score-S and AK-Score-C, respectively. Consequently, the final prediction scores are defined as follows: ${E}_{\text{AK}-\text{Score}-\text{S}}={E}_{\text{ND}}{E}_{\text{DockS}}+\alpha {E}_{\text{ATD}}$ and ${E}_{\text{AK}-\text{Score}-\text{C}}={E}_{\text{ND}}{E}_{\text{DockC}}+\alpha {E}_{\text{ATD}}$, where α is a weight parameter and was optimized to 0.65 through a grid search.

### Molecular design using MolFinder

Inhibitor candidates of ATX were generated using the MolFinder approach [41]. The approach samples novel molecules via the global optimization of a given objective function, representing desired molecular properties. The objective function was defined as the sum of predicted affinity [56], drug-likeness measured by QED [57], and the similarity to a reference molecule. The known potent inhibitor of ATX, HA-155, was used as a reference molecule for molecular generation. The binding score prediction model was trained based on the docking results obtained by docking 100,000 random molecules using AutoDock-GPU [43]. For MolFinder calculations, the number of molecules kept during optimization was set to 1000. We performed 100 independent molecular generation calculations, leading to 100,000 generated molecules. The generated molecules were docked to the ATX crystal structure and re-scored using the AK-Score scoring functions. Among the top molecules sorted by the AK-Score results, 63 molecules were selected and synthesized for validation. For an efficient validation experiment, the final 63 molecules were selected based on their synthesizability. Medicinal chemistry experts carefully investigated the structures of the top-scored molecules and selected the ones that could be readily synthesizable within two months. The synthesized molecules were tested for their interaction with ATX using a kinetic-based assay.

### FS-3-based autotaxin inhibitor screening assay

A kinetic-based ATX inhibitor assay was used to determine the inhibitory activity of the designed compounds using fluorescence excitation through cleavage of phospholipid bonds in the artificial substrate FS-3 (echelon, Salt Lake City, Utah, USA). To evaluate the % inhibition and IC_50_ values of compounds, test compounds (10 mM, 100% DMSO) were diluted ten times with the reaction buffer in pp-384 well plates (Greiner, 781,280). ATX stock (250 nM) was diluted 100 times with the reaction buffer. Reaction buffer supplemented with 224.0 mM NaCl, 80.0 mM Tris–HCl (pH 8.0), 8 mM KCl, 1.6 mM MgCl_2_, 1.6 mM CaCl_2_, 1.6 mg/mL fatty acid-free BSA. ATX and FS-3 substrates were used at a final concentration of 1 nM and 0.5 μM, respectively. The total reaction volume was 25 μL and test compounds were preincubated with ATX enzyme at 10 min before the addition of substrate. Kinetic fluorescence signals were measured every 1 min using an Envision (PerkinElmer Waltham, MA, USA) with a kinetic fluorescence readout option. The total reaction time was 30 min, 485 nm for excitation and 528 nm for emission.

## Results and discussion

### Assessment of screening accuracy with the CASF-2016 benchmark set

The CASF-2016 dataset’s benchmark outcomes clearly illustrate the effectiveness of our approach. The combinations of the binary classification model (AK-Score-NonDock) and the regression models (AK-Score-DockS and AK-Score-DockC), along with the addition of the physics-based scoring function (AK-Score-S and AK-Score-C), surpass other models in both forward and reverse screening tasks (Table 2). For the forward screening of hit compounds, AK-Score-S and AK-Score-C outperformed the other tested methods. The top 1% enrichment factors of AK-Score-C and AK-Score-S are 32.7 and 32.0, indicating that our scoring functions can enhance the efficiency of hit selection more than 30 times than random selection, corresponding to conventional high-throughput screening. These values are almost five times higher than those achieved by AutoDock-GPU and AutoDock Vina, 6.6 and 7.7. The next best top 1% enrichment factor was obtained with GenScore [58], 28.1. The other DL-based scoring functions have top 1% EF values ranging from 15.5 to 24.9, which are lower than those of AK-Score-C and AK-Score-S. Still, these values are significantly higher than those of AutoDock-GPU and AutoDock Vina. These improved EF values suggest that virtual screening via rescoring with the DL-based scoring models can be more efficient than using the conventional physics-based scoring functions.

**Table 2:** Benchmark results on the CASF2016 dataset

Model	Forward screening		Reverse screening	Scoring	Ranking	Docking
	Average EF Top 1%	Success rate (%) Top 1%	Success rate (%) Top 1%	Pearson’s $r$	Spearman’s $\rho$	Success rate (%) Top 1
AutoDock-GPU [43]	6.6	22.8	18.6	0.597	0.447	75.8
AutoDock Vina [12]	7.7	29.8	13.7	0.604	0.528	90.2
OnionNet-SFCT [55]	15.5	–	–	–	–	93.7
PIGNet (single) [35]	18.5	50.0	–	0.749	0.668	85.8
PIGNet (ensemble) [35]	19.6	55.4	–	0.761	0.682	87.0
RTMScore [36]	28.0	66.7	37.6	0.455	0.529	97.3
DeepDock [37]	16.4	43.9	23.9			87
IGModel [60]	19.4	66.7		**0.831**	**0.723**	**97.5**
GenScore [58]	28.1	71.4	32.7	0.773	0.659	96.6
DeepRMSD + Vina [61]	21.9	47.4	–	–	–	94.4
PIGNet2 [62]	24.9	66.7	–	0.747	0.651	93.0
AK-Score-NonDock	18.6	21.1	30.9	0.059	0.117	46.0
AK-Score-DockS	6.0	12.3	8.8	0.643	0.577	64.2
AK-Score-DockC	12.1	24.6	19.3	0.696	0.511	66.3
AK-Score-NonDock × AK-Score-DockS	25.7	47.4	37.2	0.356	0.391	73.0
AK-Score-NonDock × AK-Score-DockC	30.5	56.1	40.7	0.390	0.336	70.9
AK-Score-S	32.0	61.4	**43.5**	0.526	0.488	82.1
AK-Score-C	**32.7**	**71.9**	43.2	0.531	0.474	83.5

The results of AutoDock Vina were adopted from the CASF2016 dataset [13]. The results of the other models were adopted from the corresponding references. The highest values of each column are highlighted in bold

Regarding the success rate at the top 1% level, identifying at least a single hit within the top 1% of ranked candidates, the prediction accuracies of the scoring functions show a similar pattern to that of the average enrichment factor. AK-Score-C exhibited the highest success rate of 71.9% followed by GenScore at 71.4%. The next best methods, RTMScore, AK-Score-S, IGModel and PIGNet2, showed similar success rates from 61.4 to 66.7%. Overall the success rates of DL-based scoring functions are significantly higher than those of AutoDock-GPU and AutoDock Vina, 22.8 and 29.8% respectively.

Reverse screening is also an essential task for drug repositioning and target identification. Thanks to the remarkable achievement of AlphaFold2, the high-accuracy 3D structures of all human proteome are currently available, which facilitates the application of scoring functions to the reverse screening task. Regarding reverse screening success rate at the top 1% threshold, both AK-Score-S and AK-Score-C outperformed the other DL-based scoring functions and the AutoDock scoring functions. The success rates of AK-Score-S and AK-Score-C exceeded 40, 43.5 and 43.2%, which are higher than that of the third highest value of RTMScore, 37.6%. The corresponding rates of AutoDock-GPU and AutoDock Vina are 18.6, and 13.7% respectively. These results clearly show that AK-Score-S and AK-Score-C can be highly effective tools for identifying possible hits from large virtual libraries and target identification of a given molecule.

Our benchmark results suggest that our DL-based scoring models, AK-Score-DockS and AK-Score-DockC, and the physics-based AutoDock-GPU scoring functions capture partially different structural features that are important in determining protein–ligand interactions. When the outputs of the three models are correctly combined, they appear to work in a complementary manner. It should be noted that we tried combining the information from the physics-based scoring function using MLP layers. The individual terms of the AutoDock scoring function were used as input features and processed through MLP layers.

Notably, multiplying the outputs of the classification and regression models improved the performance of the individual models. The performances of individual models are only slightly better than those of the AutoDock-scoring functions. AK-Score-NonDock showed a three-fold increase in average EF but a similar success rate. AK-Score-DockC showed a moderate increase in average EF and success rate, suggesting that our assumption on the binding affinities of incorrect binding poses, penalizing with their RMSD values, is valid and effective. However, these results imply that the outputs of straightforward binary classification and regression models may not have enough resolution power. The outputs of binary classification models tend to be sharply polarized near 0 or 1.

On the other hand, the regression models, AK-Score-DockS and AK-Score-DockC, are less efficient in identifying true hits than the classification model, AK-Score-NonDock. This may be attributed to fuzzy or slightly incorrect predictions around the threshold. Our results suggest that combining the outcomes of classification and regression models can be a viable approach for other biophysical prediction tasks. In addition, combining the information of physics-based energy/scoring functions may further enhance the prediction accuracy of capturing the interactions between biomolecules.

We selected a 5 Å threshold to balance accurate binding pocket representation with computational efficiency. Our analysis revealed that the number of non-covalent edges, which are critical for protein–ligand interactions, increased by only 13% when the cutoff was raised from 5 to 6 Å, and plateaued at higher cutoffs. In contrast, covalent edges, which are less important for ligand binding, increased more rapidly beyond 5 Å, suggesting that 5 Å sufficiently captures essential non-covalent interactions.

To evaluate how different cutoffs impact prediction performance, we re-analyzed the AK-Score models using various thresholds. The results showed that docking success rates with a 4 Å cutoff were comparable to those at 5 Å, while a 6 Å cutoff caused a modest 5–10% decrease in accuracy (Supplementary Fig. 2). Similarly, enrichment factors decreased slightly with 4 and 6 Å cutoffs (Supplementary Fig. 3), which is partially expected since the current models are trained and optimized with the 5 Å cutoff. Overall, the AK-Score models demonstrated notable robustness against cutoff variations, with the 5 Å threshold providing a decent balance between prediction performance and computational efficiency.

### Assessment of screening accuracy with the DUD-E set

To investigate the generalizability of our scores, we also assessed the screening accuracy using the additional benchmark set, the DUD-E set [38]. For rigorous benchmarking, the overlap between the DUD-E and PDBbind sets was investigated by calculating sequence identities between the targets from each set, and ligand similarities based on Tanimoto similarity using the extended circular fingerprint with radius two. We assumed that two targets are similar if their sequence identity is over 90% and maximum similarities between two sets of ligands is over 0.8. Among 102 targets in the DUD-E set, 10 and 25 targets were similar to those of CASF-2016 and the training set, respectively. These results suggest that the DUD-E set is partially overlapped with PDBbind in terms of target sequence similarity and can be used as an additional test set by considering this overlap. Thus, we analyzed the benchmark results using the entire DUD-E set and after excluding highly similar targets with the training set.

The benchmark results demonstrate that AK-Score-C and AK-Score-S outperform AutoDock-GPU and OnionNet-SFCT+Vina in screening accuracy, but less than RTMScore (Table 3). The trends in improvements in EFs are consistent with those observed in the CASF-2016 benchmark set. AK-Score-C shows better performance than AK-Score-S and the other subnetwork prediction results without adding the physics-based scoring function across all EF threshold levels. Overall, the products of the classification and regression models show better performance than the single models. With a threshold of 0.5%, the average enrichment factor of AutoDock-GPU, 11.5, is almost tripled with AK-Score-C, 30.0. With thresholds of 1 and 5%, the average enrichment factors of AK-Score-C are 23.1 and 8.4, while those of AutoDock-GPU are 9.1 and 4.4. The average EF of OnionNet-SFCT+Vina at the top 1% threshold was reported to be 15.5 with the DUD-E set.

**Table 3:** A comparison of the average enrichment factors of the DUD-E benchmark set evaluated using various scoring functions at the thresholds of top 0.5, 1, and 5%

Model	Average enrichment factor		
	Top 0.5%	Top 1%	Top 5%
AutoDock-GPU [43]	11.5	9.1	4.4
OnionNet-SFCT+Vina [55]	–	15.5	–
RTMScore [36]	42.5	35.1	10.9
AK-Score-NonDock	13.9	11.6	5.9
AK-Score-DockS	11.1	9.4	5.4
AK-Score-DockC	18.1	14.6	7.1
AK-Score-NonDock × AK-Score-DockS	23.0	17.9	7.3
AK-Score-NonDock × AK-Score-DockC	27.1	21.3	7.8
AK-Score-S	25.6	20.2	8.0
AK-Score-C	30.0	23.1	8.4
AK-Score-S*	24.6	19.8	7.9
AK-Score-C*	29.4	22.8	8.4

The benchmark results after excluding targets with high sequence similarities to those in PDBbind are indicated with asterisks (*)

After excluding similar targets to the training set, similar improvements in screening accuracy were maintained. The average EFs of AK-Score-S and AK-Score-C with a threshold of 0.5% showed slight decreases by 1.0 and 0.6 respectively. At the threshold of 1.0%, the values dropped only by 0.4 and 0.3. These results indicate that improvements in EFs of AK-Score models are barely over-estimated by similarities between the training set and the benchmark set.

Since AK-Score-C consistently outperforms AK-Score-S in CASF-2016 and DUD-E benchmarks, we selected AK-Score-C as the final model for AK-Score2. These results suggest that the different types of neural networks and physics-based energy functions may capture different characteristics of protein–ligand interaction and complement each other. Thus, a proper combination of predictions from different types of models may lead to enhanced results, which is conceptually similar to an ensemble approach.

We also investigated whether the enhanced prediction accuracy by combining the ML-based and physics-based scoring functions could be generalized, i.e., the enhancement does not depend on the choice of the empirical physics-based scoring function. The identical hit screening calculation was performed with the DUD-E set using the Glide-SP scoring function [17], and the resulting average enrichment factors were evaluated (Table 4). Like the AutoDock-GPU scoring function, combining the deep-learning models: AK-Score-NonDock, AK-Score-DockS, and AK-Score-DockC with the Glide scoring function showed enhanced screening performance. However, the degree of enhancement is less significant than the AutoDock-GPU scoring function. When combined with the AK-Score-NonDock and AK-Score-DockC model, denoted as AK-Score-C-Glide, the average enrichment factor of the Glide scoring function increased from 29.4 to 34.1 with the top 0.5% threshold. Similar increases in average enrichment factor were observed with the top 1 and 5% thresholds. The combination with the AK-Score-NonDock and AK-Score-DockS model, AK-Score-S-Glide, also showed improved screening performance, but their differences were marginal. Overall, the consistent enhancements in enrichment factors demonstrate that our suggested strategy can be general and benefit other physics-based scoring functions.

**Table 4:** A comparison of average enrichment factors of the DUD-E benchmark set evaluated with Glide-SP and its combination with AK-Score models

Model	Average enrichment factor		
	Top 0.5%	Top 1%	Top 5%
Glide-SP	29.4	23.6	9.2
AK-Score-S-Glide	30.5	24.0	9.4
AK-Score-C-Glide	**34.1**	**26.9**	**9.7**

The highest values of each column are highlighted in bold

### Assessment of screening accuracy with the LIT-PCBA benchmark set

Although the CASF-2016 and DUD-E sets are considered standard benchmark sets for predicting protein–ligand interaction, there are possible concerns about the sets’ biases and generalizability. The LIT-PCBA benchmark set, consisting of 15 targets with high-quality crystal structures and many up-to-date assay data, has been suggested to address these issues. We evaluated our models’ screening power using the LIT-PCBA dataset for more rigorous validation of our models’ generalizability. By using the identical criteria with the DUD-E set, the overlap between LIT-PCBA and CASF-2016 was calculated. Among 15 targets from LIT-PCBA, two targets, ESR1 antagonist and PPAR-γ, were identified to be overlapped the training set.

**Fig. 2:**
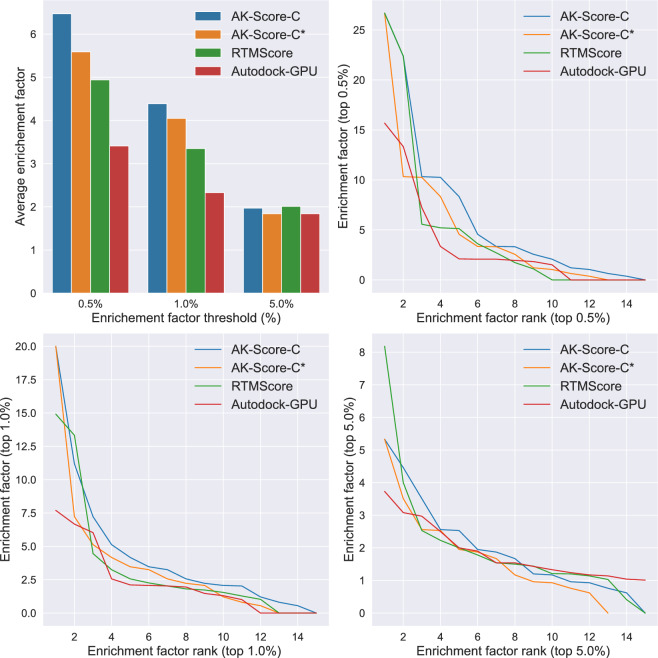
A comparative analysis of enrichment factors for the top-scored ligands across the 15 targets of LIT-PCBA benchmark set, using AK-Score-C (*blue*), RTMScore (*green*), and AutoDock-GPU (*red*). AK-Score-C* (*orange*) represents results of 13 targets, excluding overlapping targets with the training set. The average EFs at thresholds of 0.5, 1.0, and 5.0% are depicted (*top left*). The distributions of enrichment factors of the tested methods are illustrated in descending order at thresholds of 0.5% (*top right*), 1.0% (*bottom left*), and 5.0% (*bottom right*)

The screening performance assessed on LIT-PCBA demonstrates that AK-Score-C outperformed RTMScore and AutoDock-GPU across all examined thresholds (Fig. 2). Notably, this enhancement is accentuated under more stringent selection thresholds. At the 0.5 and 1.0% thresholds, AK-Score-C achieved the average EFs of 6.5 and 4.4. When the overlapping targets with the training set were removed, the corresponding average EFs decreased slightly, 5.6 and 4.1. These values are higher than the corresponding EFs of RTMScore, 4.9 and 3.4. A target-wise comparison of EFs clearly shows that AK-Score-C has better screening power on average. At the 0.5% threshold, AK-Score-C has higher EF than RTMScore in 12 targets except for the two most successful cases and the worst case. A similar trend was also observed at the 1.0% threshold; AK-Score-C outperformed RTMScore except for two targets. At the 5.0% threshold, AK-Score-C, RTMScore, and AutoDock-GPU showed almost identical performances of EFs ranging from 1.8 to 2.0.

### Ablation test

To evaluate and understand the performance of our models, we conducted a systematic ablation study. First, we ablated individual node and edge features to determine their relative importance in distinguishing true from false protein–ligand complex structures. Notably, the pharmacophore feature, which classifies the heavy atoms of proteins and ligands into seven categories (aromatic, ring, hydrophobic, hydrogen bond donor, hydrogen bond acceptor, acidic, and basic), emerged as the most critical determinant. Ablation of the pharmacophore feature led to a dramatic decrease in predictive power, with the model’s average enrichment factors in forward and reverse screening dropping by more than 45 ~ 50% (Supplementary Fig. 2 and 3). Similarly, ablating the pharmacophore feature led to the decrease in the correlation coefficient between experimental and predicted binding affinities by 0.5, resulting in almost no correlation (Supplementary Fig. 4).

These results suggest that classifying heavy atoms of proteins and ligands into seven pharmacophore types may be enough to describe protein–ligand interactions, and the relative geometries between these seven pharmacophore types play a key role in discriminating between true and false binding poses. The second and third most important features for forward screening were the number of valence electrons and atom type, both of which represent the chemical properties of heavy atoms.

In contrast to node features, edge features were less influential in predicting protein–ligand interactions overall. Among them, the distance between heavy atoms was the most significant. When this distance information was ablated, the average enrichment factor decreased from 30.5 to 7.9 for the combination of AK-Score-NonDock and AK-Score-DockC models (Supplementary Fig. 2). These findings indicate that incorporating distance information at higher resolution, such as finer binning of interatomic distances or using multiple radial basis functions, could further enhance the model’s accuracy. The overall patterns observed in the ablation test were consistent across both the AK-Score-DockC and AK-Score-DockS models, suggesting the robustness of these findings.

### Experimental application of AK-Score2 to hit-discovery process

Based on the improved screening power of AK-Score2, i.e., AK-Score-C, we applied the model to an experimental drug-discovery process. To validate this premise, we designed novel hit candidates for autotaxin [40] (ATX) inhibition and screened them using AK-Score2, AutoDock-GPU, and Glide. First, we generated more than 100,000 novel molecules using MolFinder [41]. The designed molecules were docked to the crystal structure of ATX, and the docked conformations were re-scored using AK-Score2. Among the top-scored molecules, 63 were selected and synthesized, and their binding affinities were measured using *kinetic* analysis methods. Among the 63 synthesized candidates, 23 were identified to be active, i.e., had IC_50_ values lower than 1.0 μΜ, corresponding to a success rate of 36.5%.

**Fig. 3:**
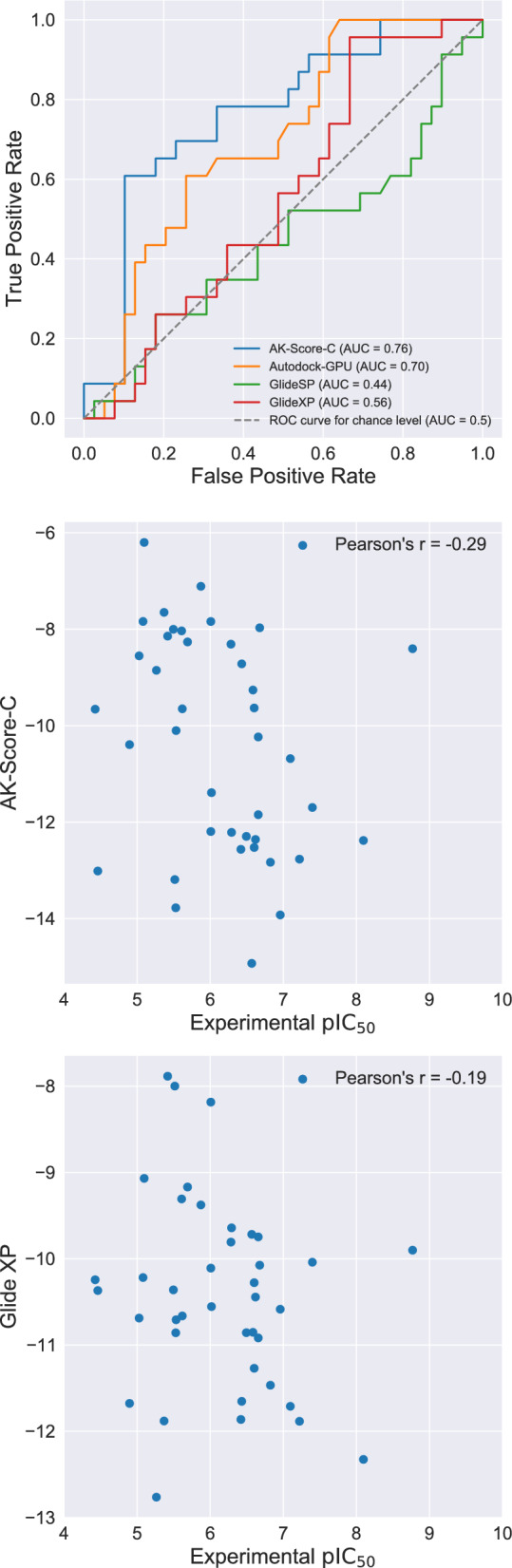
A comparison of hit discrimination performances on autotaxin using AK-Score-C, AutoDock-GPU, and Glide (*left*). The comparisons between the experimental pIC_50_ and predicted binding affinities using AK-Score-C (*middle*) and Glide (*right*)

A comparison of receiver operating characteristic (ROC) curves demonstrates that AK-Score2 identifies hit molecules of ATX efficiently (Fig. 3). The ROC curve displays how well a given scoring function discriminates active and inactive molecules and whether lower-scored molecules are prone to be active. The ROC curve of the AK-Score2 results has the highest area under the curve (AUC) value of 0.76, followed by the AutoDock-GPU and Glide-XP with AUC values of 0.70 and 0.56. Additionally, AK-Score2 scores correlate well with experimental values with a Pearson correlation coefficient of −0.29. The high hit rates, robust AUC value of 0.76, and a correlation with experimental IC_50_ values of AK-Score2 indicate that re-scoring docking results with AK-Score2 can improve the accuracy of hit-selection significantly.

## Conclusion

We developed AK-Score2, a new protein–ligand interaction prediction model, which evaluates the qualities of protein–ligand docked conformations accurately. Our model consists of three scoring functions: a structure-based binary classification model, a binding affinity regression model, and a physics-based protein–ligand scoring function. The major bottleneck of developing protein–ligand binding affinity prediction methods is the limited number of native protein–ligand complexes. The binary classification model was trained to predict whether a given protein–ligand pair binds or not. The regression model was trained not only with the natives but also with decoy complexes. We used three decoy sets: conformational decoys, which are wrongly docked conformations of true binders, cross-docked decoys, which are cross-docked conformations of non-binders within PDBbind, and random decoys, which are random docked conformations of non-binders from DDS-50 and PDBbind. The binding affinities of conformational decoys were penalized based on their RMSD from crystal structures, while the affinities of cross-docked and random decoys were set to be weaker than the threshold, −5.0 kcal/mol. While training the models, loss updates were performed selectively for the native and decoy sets.

The screening benchmark results with the CASF-2016, DUD-E, and LIT-PCBA sets demonstrate that AK-Score-C screens hit candidates more accurately than the existing ML-based and physics-based models. For the CASF-2016 and LIT-PCBA sets, AK-Score2 yielded higher EFs than RTMScore and AutoDock-GPU in most examined targets. For the DUD-E set, AK-Score2 showed better performance than AutoDock-GPU and OnionNet-SFCT+Vina [51], but worse performance than RTMScore. Our results demonstrate that training the classification and regression models separately, then combining their outputs, may be an effective strategy for predicting intermolecular interactions. It would also be worth investigating whether combining the ML-based and physics-based scoring functions may apply to predictions of other biomolecules, such as protein–protein interactions.

Additionally, combined with the novel molecular generation algorithm, MolFinder, AK-Score2 successfully discriminated novel hit candidates for ATX inhibition. Novel ATX inhibition candidates designed with MolFinder were scored with AK-Score2, and the top 63 candidates were synthesized. Among the synthesized molecules, 23 have p*K*_d_ values higher than 6. The results show that AK-Score2 facilitates hit screening and lead optimization, which can eventually accelerate drug discovery. We believe that the proposed model architecture and training procedure can be applied to other ML models related to drug discovery.

## Supplementary Information

Additional file 1.

## Data Availability

All protein–ligand conformations used in model training and test are available at https://zenodo.org/doi/10.5281/zenodo.10596973. The source code to perform inference using AK-Score2 model is available at the following GitHub repository: https://github.com/arontier/Akscore2_Paper/tree/main.
