# Supplementary material for: Accurate prediction of protein–ligand interactions by combining physical energy functions and graph-neural networks
Source: J Cheminform. 2024 Nov 4;16:121. doi: 10.1186/s13321-024-00912-2 (PMC11536843; doi:10.1186/s13321-024-00912-2)
Supplement: Supplementary file 1 — Additional file 1. [file 13321_2024_912_MOESM1_ESM.docx]

**Supplementary Information**

**Supplementary Figure**


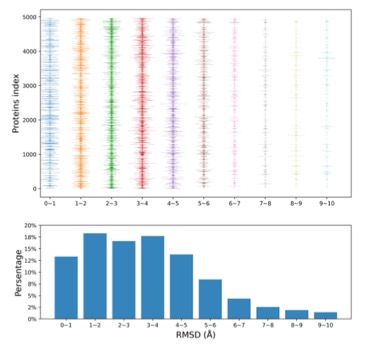


**Figure 1.** (a) The RMSD distributions of docked conformations of proteins in the training set using Autodock-GPU. The x-axis corresponds to the RMSD values from the crystal structures. The y-axis represents protein indices of the training set. The lengths of horizontal lines are proportional to the number of sampled docked conformations corresponding to a RMSD bin. (b) The RMSD distribution of docked conformations of the protein-ligand pairs in the training dataset, $\mathcal{D}_{\mathrm{native}}$ and $\mathcal{D}_{\mathrm{conf}}$, obtained with Autodock-GPU.

Figure 2. The change of forward screening success rate by feature ablation. X-axis represents an ablated feature, and Y-axis corresponds to the change of success rate compared to the original model. Feature ablation was performed by making the corresponding node or edge feature zero,

Figure 3. The change of reverse screening success rate by feature ablation. X-axis represents an ablated feature, and Y-axis corresponds to the change of success rate compared to the original model.

Figure 4. The change of Pearson correlation coefficient between experimental and predicted binding affinities by feature ablation. X-axis represents an ablated feature, and Y-axis corresponds to the change of Pearson correlation coefficient between experimental and predicted binding affinities compared to the original model.

**Supplementary Tables**

Table S1. Node features

| **Feature (size)** | **list** |
| --- | --- |
| Atom type (20) | C, H, O, N, S, P, F, Cl, Br, I, B, Se, Fe, Ru, Mn, Co, Ni, Cu, Zn, other (one-hot) |
| Degree of atom (7) | 0, 1, 2, 3, 4, 5, 6 (one-hot) |
| Num Hs (5) | 0, 1, 2, 3, 4 (one-hot) |
| Implicit Valence (6) | 0, 1, 2, 3, 4, 5 (one-hot) |
| Explicit Valence (6) | 0, 1, 2, 3, 4, 5 (one-hot) |
| Implicit Hs (5) | 0, 1, 2, 3, 4 (one-hot) |
| Explicit Hs (2) | 0, 1 (one-hot) |
| Hybridization (5) | sp, sp2, sp3, sp3d, sp3d2 (one-hot) |
| Radical (3) | 0,1,2 (one-hot) |
| Formal charge (7) | -2, -1, 0, 1, 2, 3, 4 (one-hot) |
| Pharmacophore (7) | Aromatic, Ring, Hydrophobic, H-bond donor, H-bond acceptor, Acidic, Basic (binary) |

Table S2. Edge features

| **Edge type** | **Feature (size)** | **list** |
| --- | --- | --- |
| Covalent bond | Bond type (4) | Single, double, triple, aromatic (one-hot) |
|  | Stereo type (6) | Any, Cis, Trans, E, Z, none (one-hot) |
|  | Ring (1), Conjugating (1) | Binary |
| Non-covalent bond | PL bond type (5) | Hydrophobic, PL Hydrogen, LP Hydrogen, PL ionic, LP ionic (binary) |
|  | Distance (4) | <2 Å, <4 Å, <6 Å, <8 Å (binary) |
|  | Atom-Atom connection (3) | Protein-Protein, Ligand-Ligand, Protein-Ligand (one-hot) |

Table S3. Detailed hyperparameters of network structure of the models used in this paper.

|  | Akscore2_NonDock | Akscore2_DockS | Akscore2_DockC |
| --- | --- | --- | --- |
| Initial node  feature dimension | 73 | 73 | 73 |
| Edge  feature dimension | 12 | 24 | 24 |
| Embedding layer | In channel: 73  Out channel: 256 | In channel: 73  Out channel: 256 | In channel: 73  Out channel: 256 |
| GNN | GNN-Protein: {  layer: 5  In channel: 256  Out channel: 256  }  GNN-Ligand: {  layer: 3  In channel: 256  Out channel: 256  } | GNN-Protein Ligand Complex: {  layer: 5  In channel: 256  Out channel: 256  } | GNN-Protein Ligand Complex: {  layer: 5  In channel: 256  Out channel: 256  } |
| MLP | Linear Layer 1: {  In channel: 512  Out channel: 512  }  Linear Layer 2: {  In channel: 512  Out channel: 256  }  Linear Layer 3: {  In channel: 256  Out channel: 1  } | Linear Layer 1: {  In channel: 256  Out channel: 256  }  Linear Layer 2: {  In channel: 256  Out channel: 128  }  Linear Layer 3: {  In channel: 128  Out channel: 1  } | Linear Layer 1: {  In channel: 256  Out channel: 256  }  Linear Layer 2: {  In channel: 256  Out channel: 128  }  Linear Layer 3: {  In channel: 128  Out channel: 1  } |

**Supplementary Methods**

**Node and edge feature construction approach**

Molecule and protein represented graphs with atoms and two valence bonds. Properties of atoms are represented as 73 node features (Table S1), and properties of bonds are represented as 24 edge features (Table S2). First, Edge connect type is used to express whether the binding is within the ligand, within the protein, or between the ligand and the protein. There are 20 of atom type (C, H, O, N, S, P, F, Cl, Br, I, B, Se, Fe, Ru, Mn, Co, Ni, Cu, Zn, other) are represented as one-hot vectors. Various other atomic properties were obtained using RDKit and represented as one-hot and binary.

There are five features (Hydrophobic, H-Donor, H-Accepter, Acidic, Basic) that were not obtained with RDKit, among which hydrophobic is expressed by dividing the charge of each atom calculated by Autodock Tools (MGLTools) by 0 if it is smaller than 0.2 and by 1 if it is larger. H donors and H acceptors are represented by 0 and 1 according to the number of Hs possessed by N, O, S, and P atoms, acidic and basic are represented by 0 and 1 for carboxyl and amino groups. The types of bonds used were covalent bonds within the ligand and protein and non-covalent bonds with interactions between atoms of the ligand and atoms of the protein. Edge features were represented by one-hot vectors according to binding type, stereotype, ring, conjunction, distance. Covalent bonds are represented by one-hot vectors in four types (single, double, triple, and aromatic), and in five steric directions (Any, Cis, Trans, E, Z, and None). Then checked Ring and Conjugation. When the atomic distance between the ligand atom and the protein atom is within 8 Å, a non-covalent bond between the two atoms is created. There are two types of non-covalent bonds: PL bond type, and distance. PL bond type have hydrophobic, hydrogen bond, and ionic bond. It is a hydrophobic bond if both atoms of the bond have hydrophobic. If an atom has one H accepter and one H donor of each other, it is hydrogen bond. Similarly, if each has acidic and basic, it is ionic bond. Hydrogen bond and ionic bond are distinguished from ligand to protein or from protein to ligand. Distances between ligands and protein atoms are represented by four thresholds, less than 2 Å, 4 Å, 6 Å and 8 Å.
